# Real-world outcomes of stage III non-small cell lung cancer in the durvalumab era: insights from a socioeconomically deprived population

**DOI:** 10.1016/j.ctro.2026.101232

**Published:** 2026-07-10

**Authors:** Adam L. Peters, Harrison Douglas Stubbs, Sean F. Duncan, Audrey Shaw, Karen Moore, Erin McGarry, Selina Tsim, Graeme Lumsden, Mark Stares, Iain Phillips, David Y. Lewis, Kevin G. Blyth, John D. Maclay

**Affiliations:** a Beatson West of Scotland Cancer Centre, Glasgow, Scotland, UK; b School of Cancer Sciences, University of Glasgow, UK; c Department of Respiratory Medicine, Glasgow Royal Infirmary, Glasgow, Scotland, UK; d Cancer Research UK Scotland Centre, Institute of Genetics and Cancer, University of Edinburgh, Edinburgh, UK; e Department of Respiratory Medicine, Queen Elizabeth University Hospital, Glasgow, UK

## Abstract

**Background:**

The introduction of consolidation durvalumab after chemoradiotherapy (CRT) for unresectable stage 3 non-small cell lung cancer (NSCLC), as established by the PACIFIC trial, has transformed survival outcomes. However, its impact remains uncertain in populations characterised by high levels of socioeconomic deprivation, multimorbidity, and historically poorer cancer outcomes compared to other regions.

**Materials/methods:**

This retrospective observational cohort study included all patients with stage III unresectable NSCLC who received radical (chemo)radiotherapy, between 01/01/2017 – 31/12/2022. Clinical outcomes were compared across treatment groups: radiotherapy alone, sequential CRT, concurrent CRT, and concurrent CRT followed by consolidation durvalumab. Multivariate Cox proportional hazards regression was employed to identify factors associated with outcomes.

**Results:**

656 patients with stage III NSCLC treated with radical radiotherapy were included. The median age was 69 years, and 54% were male. Most patients had a PS of 0 or 1 (81%), the median Charlson Comorbidity Index (CCI) was 3 (2 – 5) and 62% resided in the most deprived socioeconomic quintiles. Radiotherapy alone was the most common treatment (59%). Administration of radiotherapy alone appeared to be influenced by older age and higher performance status, and not by deprivation or objective functional measurements of multimorbidity (i.e. CCI). Reflex biomarker testing improved over time, while the use of sequential CRT declined, and the rates of concurrent CRT, with or without durvalumab, increased.

Multivariate regression demonstrated that patients receiving concurrent CRT plus durvalumab achieved superior 2 year progression-free survival (PFS) compared with concurrent CRT alone (HR 0.51, 0.33 – 0.78, *p*=0.002). Patients treated with radiotherapy alone had the poorest 2-year OS and PFS, independent of other confounders. Delivering a full year of durvalumab was challenging, with 55% of patients who started durvalumab being unable to complete treatment. However, 53% of those who discontinued durvalumab early remained disease-free at 2 years.

**Conclusion:**

This study reinforces the progression-free survival benefit of consolidation durvalumab in patients able to undergo concurrent CRT within a large real-world cohort. Although the cohort was socially deprived and comorbid, the high proportion of patients receiving radical radiotherapy alone, and their associated poorer survival outcomes, were not explained by this, revealing a mismatch between objective fitness and clinical decision making, The associated poorer survival outcomes from radiotherapy alone highlights the urgent need to optimise treatment strategies for such patients.

## Highlights

• Consolidation durvalumab following concurrent CRT significantly improved 2-year PFS with acceptable tolerability in a large UK real-world cohort.
• Increasing use of concurrent CRT and declining use of sequential CRT over time reflect growing confidence in delivering this treatment.
• Patients treated with radiotherapy alone, the most common modality, had the poorest outcomes, underscoring the need to evaluate its effectiveness without systemic treatment.
• The cohort demonstrated high levels of socioeconomic deprivation and multimorbidity.

## 1 Background

In 2017, the PACIFIC trial demonstrated significant survival benefits of adding consolidation durvalumab to concurrent chemoradiotherapy (CRT) for patients with unresectable, radically treatable stage III non-small cell lung cancer (NSCLC) [1]. While the trial enrolled patients with performance status (PS) 0 or 1, many individuals with locally advanced NSCLC present with compromised fitness due to disease burden and comorbidities, making delivery of this treatment challenging in routine clinical practice. Consequently, delivery of concurrent CRT is not always possible even in patients with PS 0 or 1. More recently, PACIFIC-6 reported similar benefits for patients receiving sequential CRT followed by durvalumab, offering an alternative for those unable to tolerate concurrent treatment [2].

The CODAK study, a UK-based retrospective cohort analysis, confirmed real-world survival benefits of durvalumab after CRT, comparable to PACIFIC at 12- and 24-months. Outcomes were maintained despite 29% of patients discontinuing treatment due to adverse effects [3]. While CODAK helped bridge the gap between a randomised controlled trial and clinical practice, it did not explore how PACIFIC influenced the delivery of other radical treatments for unresectable NSCLC or provide comparative data on progression free survival (PFS) and overall survival (OS) across treatment modalities in a real-world setting.

To address these gaps, we have reviewed treatment patterns for unresectable stage III NSCLC from 2017 to 2022, assessing changes over time spanning the adoption of adjuvant durvalumab, and comparing 2-year OS and PFS among patients treated with radical radiotherapy alone, sequential CRT, concurrent CRT, and concurrent CRT plus durvalumab. The primary endpoint of this study was 2-year PFS, with secondary endpoints including 2-year OS, treatment pattern evolution, and durvalumab completion/side effects.

## 2 Methods

This retrospective observational cohort study was conducted and reported in accordance with the Strengthening the Reporting of Observational studies in Epidemiology (STROBE) guidelines [4]. Consecutive patients diagnosed with stage III NSCLC in the West of Scotland (a region including 11 centres, including university teaching and district general hospitals) and treated at the Beatson Oncology Centre were included. Eligible cases were diagnosed between January 1, 2017, and December 31, 2022, and completed a course of radical radiotherapy (≥54Gy) as their first-line treatment.

Data were prospectively collected by clinical audit staff in each NHS Board through the nationally agreed Quality Performance Indicator dataset and definitions, with approval for storage and future analysis granted nationally. These data were linked with cause-of-death records from NHS National Services Scotland. Oversight of patient-identifiable data was provided by the Caldicott Guardian, and permissions for specific analyses were obtained a priori. Electronic clinical records were reviewed to confirm lung cancer recurrence within two years post-radiotherapy, defined by radiological evidence (with or without biopsy) and MDT consensus. For patients deemed to have died of other causes, records were reviewed for confirmation. Missing data were manually extracted from electronic records.

Patients were staged using the TNM Classification of Malignant Tumours, 8th Edition [5], and performance status (PS) was assessed using the Eastern Cooperative Oncology Group (ECOG) scale [6]. Patients with PS > 2 were excluded. For analyses, patients were categorised into four histopathological groups: squamous cell carcinoma, adenocarcinoma, NSCLC-other, and no histology. Survival follow-up was two years from the start of radiotherapy.

Patients were grouped into three treatment eras reflecting the progressive adoption of adjuvant durvalumab. An early access scheme began in December 2018, and formal approval by the Scottish Medicines Committee (SMC) occurred in June 2019. The **pre-durvalumab era** (2017–2018) included patients treated prior to approval, when use was rare. The **peri-durvalumab era** (2019–2020) captured the transition period following approval, as clinicians began incorporating durvalumab. The **post-durvalumab era** (2021−2022) represented routine use of durvalumab as standard of care after concurrent CRT in eligible patients (PD-L1 ≥ 1%).

### 2.1 *Statistical analyses*

Data are presented as proportions (%), mean (SD) for normally distributed variables or median (IQR) for non-normal distributions. Comparisons across time periods and treatment modalities were performed using Mann-Whitney U, Kruskal-Wallis, and Chi-squared tests, as appropriate. Survival was analysed using the Kaplan-Meier method, with a log-rank test for statistical significance. Multivariate regression analysis was conducted using the Cox proportional hazards model to evaluate associations between covariates and 2-year OS and PFS. OS was measured from the date of completion of radiotherapy to death from any cause or censoring. PFS was measured from the date of completion of radiotherapy to any recurrence (local or metastatic) of tumour, death from any cause or censoring. Survival times were administratively censored at 24 months to enable comparison of 2-year outcomes. Median follow-up was estimated using the reverse Kaplan–Meier method and was 43 months.

To enable valid comparison with PACIFIC outcomes, patients with PS 2 and those treated in the pre-durvalumab era (2017–2018) were excluded from multivariate analysis, as durvalumab was only licensed for PS 0–1 and introduced into routine practice in 2019. Forest plots and stratified survival curves were then generated from these models. Covariates included age, PS, Scottish Index of Multiple Deprivation (SIMD), Charlson Comorbidity Index (CCI), histology, stage and treatment modality. Variance Inflation Factor (VIF) scores were calculated to assess multicollinearity between the covariates, all scores were less than 1.3, indicating no multicollinearity. The proportional hazards assumption was tested using Schoenfeld residuals, with no evidence of violation (global *p* = 0.87). Analyses were performed using R v4.2.2 (Vienna, Austria). A *p*-value <0.05 was considered statistically significant.

## 3 Results

### 3.1 Study population

**Fig. 1:**
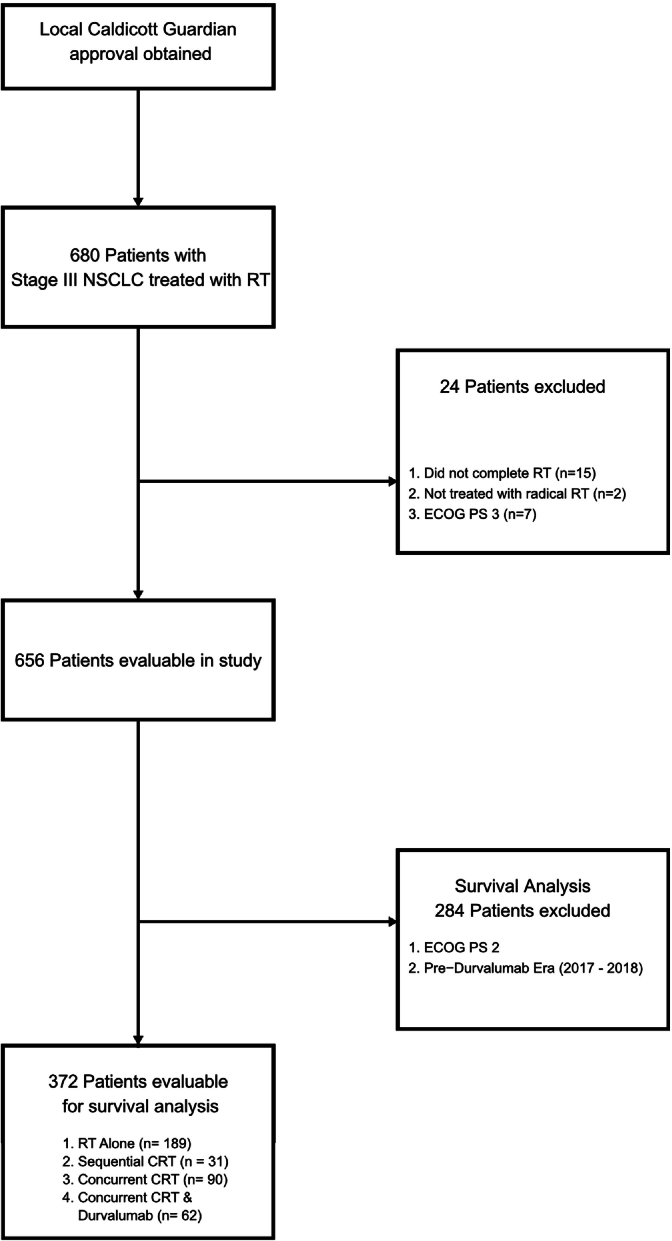
CONSORT-style flow diagram illustrating patient inclusion and exclusion (N = number of patients).

The study population (Fig. 1) included all patients with stage III unresectable NSCLC treated with radical RT. Initially, 680 patients were enrolled to the study. Following review, 24 patients were excluded leaving 656 patients evaluable in the study. Further exclusions were applied for the survival analysis specifically (284 exclusions), with 372 patients evaluable for the survival analysis.

### 3.2 Patient demographics

A total of 656 patients with stage III NSCLC treated with radical radiotherapy were included (Fig. 1). The median age was 69 years, and 54% were male. Most patients had a PS of 0 or 1 (81%), and 62% of patients resided in the most deprived socioeconomic quintiles (Scottish Index of Multiple Deprivation (SIMD) 1 or 2). Comorbidity was present in the cohort with a median CCI of 3 (2 - 5). 15% had chronic obstructive pulmonary disease (COPD), 8% cardiac disease, 6% diabetes, 5% peripheral vascular disease (PVD) and 2% liver disease. Nearly all patients (98%) were current or ex-smokers. Squamous cell carcinoma was the most common histological subtype (52%), followed by adenocarcinoma (31%) (Table 1, Table 2).

**Table 1:** Patient demographics and comorbidity data.

	Whole Cohort		Treatment Modality				
Variable	N	*N* = 656^1^	RT alone *N* = 387^1^	Sequential *N* = 59^1^	Concurrent CRT *N* = 141^1^	Concurrent CRT & Durvalumab *N* = 69^1^	p-value^2^
**Age at MDT**	656	69 (62–75)	72 (66–78)	66 (61–71)	64 (59–69)	62 (58–68)	**<0.001**
**Gender**	656						0.25
Female		299 (46)	185 (48)	26 (44)	64 (45)	24 (35)	
Male		357 (54)	202 (52)	33 (56)	77 (55)	45 (65)	
**ECOG PS**	656						**<0.001**
0		182 (28)	57 (15)	23 (39)	66 (47)	36 (52)	
1		351 (54)	220 (57)	31 (53)	70 (50)	30 (43)	
2		123 (19)	110 (28)	5 (9)	5 (4)	3 (4)	
**Smoking Status**	656						0.14
Current		301 (46)	170 (44)	22 (37)	73 (52)	36 (52)	
Ex Smoker		338 (52)	203 (52)	37 (63)	66 (47)	32 (46)	
Non Smoker		17 (3)	14 (4)	0 (0)	2 (1)	1 (1)	
**Charlson Comorbidity Index (CCI)**	656	3 (2–5)	3 (2–5)	4 (3–6)	3 (2–5)	3 (2–5)	0.20
**Scottish Index of Multiple Deprivation (SIMD)**	656						0.91
1 (most deprived)		241 (37)	142 (37)	25 (42)	52 (37)	22 (32)	
2		167 (25)	103 (27)	12 (20)	31 (22)	21 (30)	
3		108 (16)	65 (17)	9 (15)	25 (18)	9 (13)	
4		72 (11)	42 (11)	6 (10)	17 (12)	7 (10)	
5 (least deprived)		68 (10)	35 (9)	7 (12)	16 (11)	10 (14)	
**Chronic Obstructive Pulmonary Disease (COPD)**	656	100 (15)	55 (14)	10 (17)	24 (17)	11 (16)	0.84
**Cardiac Disease**	656	55 (8)	31 (8)	6 (10)	12 (9)	6 (9)	0.95
**Diabetes**	656	38 (6)	18 (5)	6 (10)	10 (7)	4 (6)	0.33
**Liver Disease**	656	13 (2)	7 (2)	1 (2)	2 (1)	3 (4)	0.51
**Cerebrovascular Disease**	656	42 (6)	22 (6)	7 (12)	8 (6)	5 (7)	0.32
**Peripheral Vascular Disease (PVD)**	656	34 (5)	18 (5)	5 (9)	10 (7)	1 (1)	0.21
**FEV1/FVC Ratio (%)**	363	63 (55–70)	63 (54–71)	59 (54–68)	65 (58–70)	65 (55–71)	0.64
**TLCO (%)**	330	64 (52–76)	62 (50–75)	59 (52–67)	68 (55–78)	65 (51–81)	0.18

1 Median (IQR); n (%)

2 Kruskal-Wallis rank sum test; Pearson's Chi-squared test

**Table 2:** Tumour and treatment details.

	Whole Cohort		Treatment Modality				
Variable	N	N = 656^1^	RT alone N = 387^1^	Sequential N = 59^1^	Concurrent CRT N = 141^1^	Concurrent CRT & Durvalumab N = 69^1^	p-value^2^
**Histology**	656						**<0.001**
Adenocarcinoma		203 (31)	102 (26)	19 (32)	51 (36)	31 (45)	
Squamous		342 (52)	217 (56)	33 (56)	63 (45)	29 (42)	
NSCLC other		76 (12)	36 (9.3)	6 (10)	25 (18)	9 (13)	
No histology		35 (5)	32 (8.3)	1 (2)	2 (1)	0 (0)	
**Stage**	656						**0.012**
IIIA		356 (54)	227 (59)	24 (41)	72 (51)	33 (48)	
IIIB		271 (41)	150 (39)	32 (54)	57 (40)	32 (46)	
IIIC		29 (4)	10 (3)	3 (5)	12 (9)	4 (6)	
**PDL1 Status**	656						**<0.001**
<1%		273 (42)	164 (42)	26 (44)	81 (57)	2 (3)	
>50%		117 (18)	55 (14)	11 (19)	16 (11)	35 (51)	
1–50%		145 (22)	73 (19)	13 (22)	27 (19)	32 (46)	
Unknown		121 (18)	95 (25)	9 (15)	17 (12)	0 (0)	
**ALK/EGFR/ROS1 Status**	656						**0.005**
EGFR positive		6 (1)	6 (2)	0 (0)	0 (0)	0 (0)	
EGFR uncertain significance		2 (1)	1 (1)	0 (0)	1 (1)	0 (0)	
Negative		228 (35)	108 (28)	21 (36)	66 (47)	33 (48)	
Not tested - SCC		340 (52)	216 (56)	33 (56)	62 (44)	29 (42)	
Unknown		80 (12)	56 (14)	5 (9)	12 (9)	7 (10)	
**RT Dose/Fractionation**	656						**<0.001**
54Gy/36# (CHART)		140 (21)	135 (35)	5 (9)	0 (0)	0 (0)	
55Gy/20#		495 (75)	252 (65)	51 (86)	132 (94)	60 (87)	
Other dose		21 (3)	0 (0)	3 (5)	9 (6)	9 (13)	
**Time on Adjuvant Durvalumab (months)**	69	9 (2−12)	NA (NA – NA)	NA (NA – NA)	NA (NA – NA)	9 (2–12)	
**Time Period**	656						**<0.001**
Pre-Durvalumab		188 (29)	111 (29)	26 (44)	47 (33)	4 (6)	
Peri-Durvalumab		252 (38)	157 (41)	21 (36)	42 (30)	32 (46)	
Post-Durvalumab		216 (33)	119 (31)	12 (20)	52 (37)	33 (48)	

1 Median (IQR); n (%).

2 Kruskal-Wallis rank sum test; Pearson's Chi-squared test.

### 3.3 Evolution of treatment and biomarker testing

**Fig. 2:**
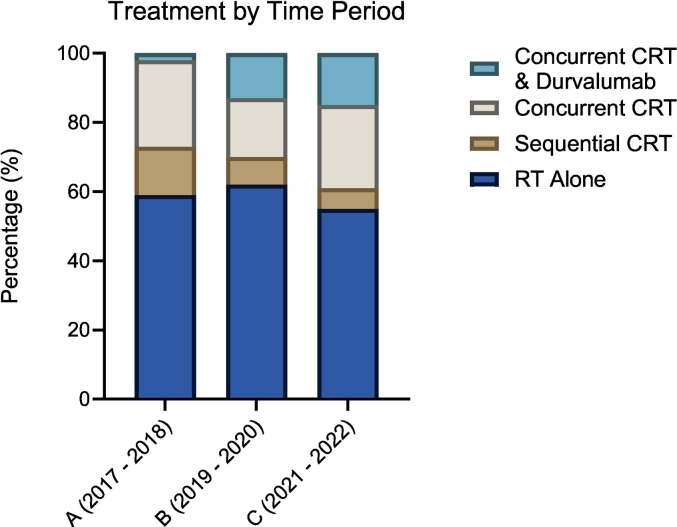
Stacked bar plot showing treatment modalities distribution across study periods: A = pre-durvalumab, B = peri-durvalumab and C = post-durvalumab.

Across the entire cohort, 59% of patients received RT alone, 22% received concurrent CRT, and 9% received sequential CRT. Concurrent CRT combined with durvalumab accounted for 11% of cases. The proportion of patients receiving concurrent CRT (with or without durvalumab) increased from 27% in 2017–2018 to 39% in 2021–2022, while sequential CRT declined from 14% to 6%. Rates of RT alone remained stable throughout the study period (Fig. 2; Supplementary Table 1).

A total of 69 patients received at least one dose of durvalumab following concurrent CRT, with a median treatment duration of 9 months. Completion of the full one-year course occurred in 45% of these (Fig. 5). We did not observe differences in 2-year OS or PFS between the three time periods.

Reflex PDL1 testing was introduced after durvalumab became available, increasing from 59% in 2017–2018 to 93% in 2021–2022. Testing for other companion biomarkers also improved (84% to 93%) and confirmatory tissue diagnosis rose from 93% to 96% over the same period.

### 3.4 Overall and progression free survival based on treatment modality

After excluding patients with PS 2 and those treated in the pre-durvalumab era, 372 patients were included in the survival analysis. Both 2-year OS and PFS were strongly associated with treatment modality (Table 3, Table 4, *p* < 0.001 & *p* = 0.005 respectively). At two years post treatment, mortality was 70% for patients receiving RT alone, compared with 46% for sequential CRT, 44% for concurrent CRT, and 29% for concurrent CRT plus durvalumab. Progression-free rates at two years were 38% for RT alone, 41% for sequential CRT, 39% for concurrent CRT, and 64% for concurrent CRT plus durvalumab. Among patients completing 12 months of durvalumab, 77% remained recurrence-free at two years.

**Table 3:** Survival data for whole cohort.

	Whole Cohort		Treatment Modality				
Variable	N	N = 656^1^	RT alone N = 387^1^	Sequential N = 59^1^	Concurrent CRT N = 141^1^	Concurrent CRT & Durvalumab N = 69^1^	p-value^2^
**Deceased at 2-years**	656	381 (58)	272 (70)	27 (46)	62 (44)	20 (29)	**<0.001**
**Lung Cancer Death at 2-years**	656	336 (51)	237 (61)	23 (39)	59 (42)	17 (25)	**<0.001**
**Recurrence at 2-years**	656	385 (59)	239 (62)	35 (59)	86 (61)	25 (36)	**0.001**
**Local recurrence at 2-years**	656	159 (24)	102 (26)	15 (25)	35 (25)	7 (10)	**0.037**
**Distant recurrence at 2-years**	656	239 (36)	146 (38)	21 (36)	54 (38)	18 (26)	0.30

1 Median (IQR); n (%).

2 Kruskal-Wallis rank sum test; Pearson's Chi-squared test.

**Table 4:** Survival cohort.

	Whole Cohort		Treatment				
Variable	N	*N* = 372^1^	Concurrent CRT *N* = 90^1^	Concurrent CRT & Durvalumab *N* = 62^1^	Sequential *N* = 31^1^	RT alone *N* = 189^1^	p-value^2^
**Deceased at 2 years**	372	204 (55)	39 (43)	19 (31)	14 (45)	132 (70)	**<0.001**
**2-year OS Hazard Ratio**	372		–	0.87, *p* = 0.54	1.56, *p* = 0.086	2.35, **p** **<** **0.001**	
**Lung Cancer Death at 2 years**	372	177 (48)	37 (41)	16 (26)	12 (39)	112 (59)	**<0.001**
**Recurrence at 2 years**	372	213 (57)	54 (60)	23 (37)	18 (58)	118 (62)	**0.005**
**2-year PFS Hazard Ratio**	372		–	0.50, **p** **=** **0.002**	1.16, *p* = 0.053	1.34, *p* = 0.063	
**Local Recurrence at 2 years**	372	79 (21)	17 (19)	7 (11)	8 (26)	47 (25)	0.12
**Distant Recurrence at 2 years**	372	145 (39)	40 (44)	16 (26)	11 (35)	78 (41)	0.10
**Received Treatment on Recurrence**	221	120 (54)	42 (75)	19 (79)	11 (61)	48 (39)	**<0.001**
**Type of Treatment on Recurrence Received**	120						**0.005**
Chemotherapy		21 (18)	4 (10)	6 (32)	1 (9)	10 (21)	
Immunotherapy		53 (44)	28 (67)	4 (21)	9 (82)	12 (25)	
Palliative RT		34 (28)	7 (17)	5 (26)	1 (9)	21 (44)	
SRS/SABR		4 (3)	1 (2)	1 (5)	0 (0)	2 (4)	
Surgery		5 (4)	2 (5)	2 (11)	0 (0)	1 (2)	
Targeted therapy		3 (3)	0 (0)	1 (5)	0 (0)	2 (4)	

1 n (%).

2 Pearson's Chi-squared test.

At recurrence, only 39% of patients treated with RT alone received further therapy, compared with 75% for concurrent CRT, 79% for concurrent CRT plus durvalumab, and 61% for sequential CRT (*p* < 0.001, Table 4). Immunotherapy was the most common subsequent treatment for patients previously treated with CRT without adjuvant durvalumab, whereas palliative RT was most frequent among those initially treated with RT alone.

**Fig. 3:**
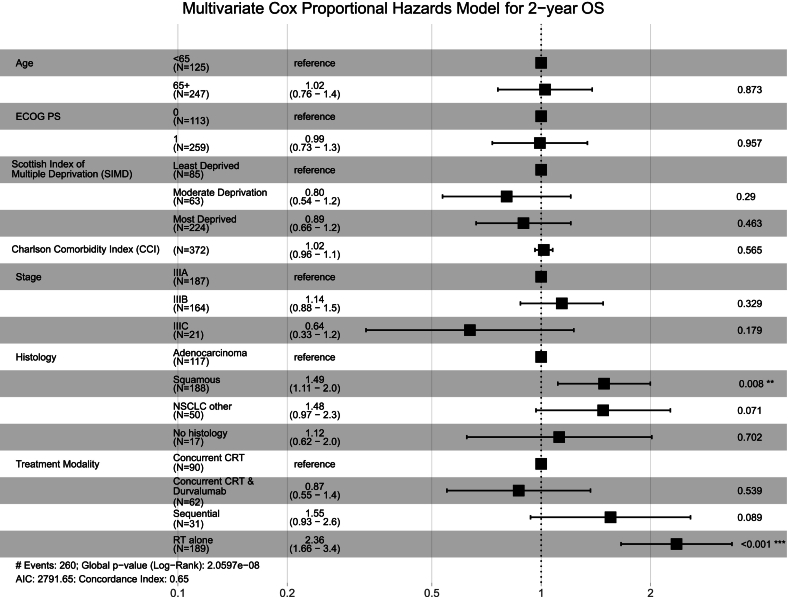

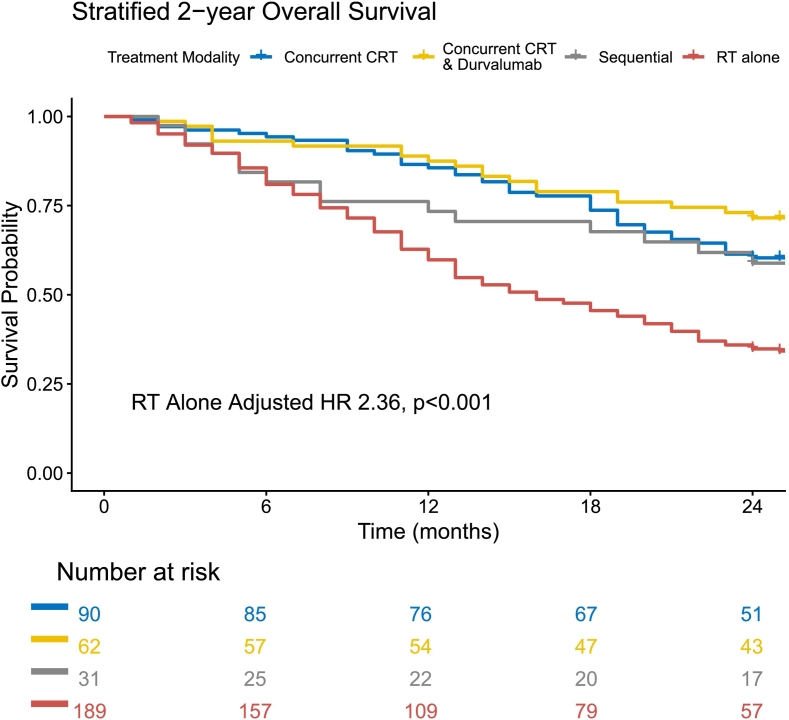
A, Forest plot showing results of the multivariate Cox proportional hazards model for 2-year overall survival (OS). B, Adjusted Kaplan-Meier survival curves for 2-year OS.

**Fig. 4:**
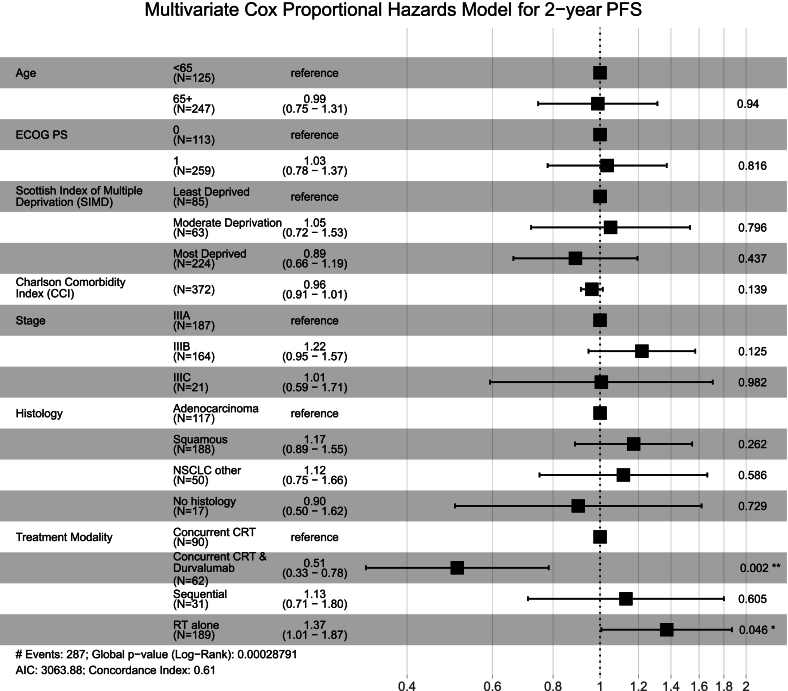

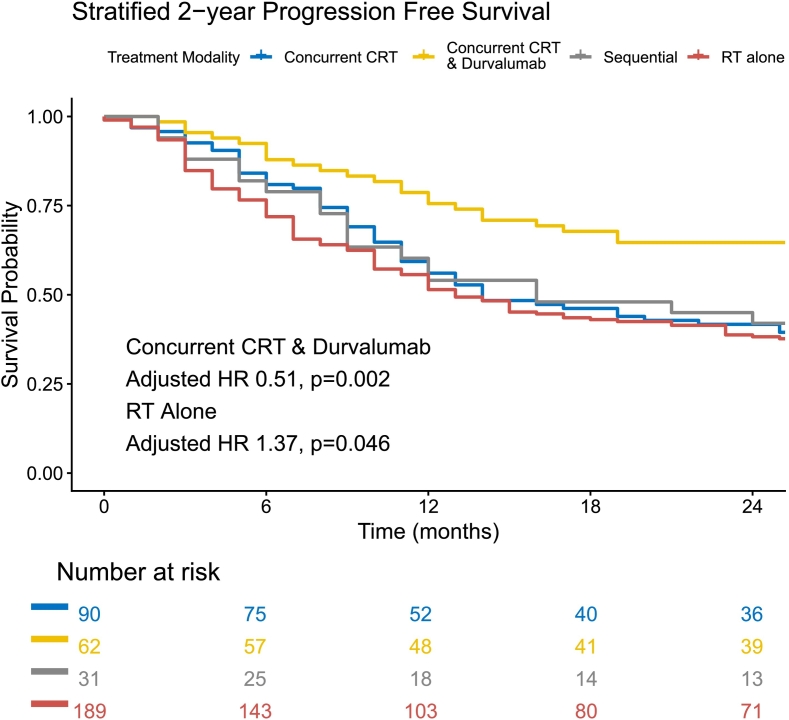
A, Forest plot showing the results of the multivariate Cox proportional hazards model for 2-year progression free survival (PFS). B, Adjusted Kaplan-Meier survival curves for 2-year PFS.

Multivariate Cox regression identified squamous histology (HR 1.49, 95% CI 1.11–2.0, *p* = 0.008) as an independent predictor of poorer OS compared to adenocarcinoma (Fig. 3A & B). There were no differences between concurrent CRT and concurrent CRT & durvalumab, however RT alone was associated with significantly poorer 2-year OS compared to concurrent CRT (HR 2.36, 95% CI 1.66–3.4, *p* < 0.001) (Fig. 3A & 3B). Sequential CRT trended towards poorer 2-year OS compared to concurrent CRT, but this was non-significant (HR 1.55, 95% CI 0.92–2.6, *p* = 0.089). While 2-year OS was similar between concurrent CRT alone and concurrent CRT plus durvalumab, the addition of durvalumab conferred a marked improved in 2-year PFS (HR 0.51, 95% CI 0.33–0.78, *p* = 0.002) compared with concurrent CRT (Fig. 4A & B). RT alone had the poorest 2-year PFS outcomes (HR 1.37, 95% CI 1.01–1.87, *p* = 0.046).

### 3.5 Treatment side effects

**Fig. 5:**
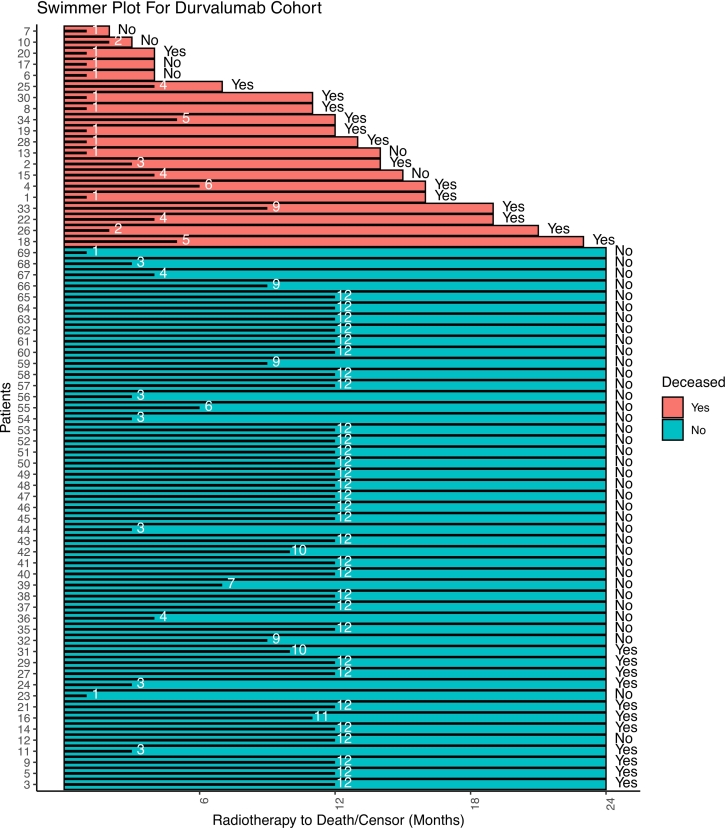
Swimmer plot of the durvalumab cohort. Each bar represents an individual patient; black segments within bars indicate time on durvalumab (in months), with white text showing duration. Text at the end of bars denotes recurrence status at 2 years.

Among the 69 patients treated with concurrent CRT plus durvalumab, 38 (55%) did not complete the planned treatment course (Fig. 5). Side effects were the most common reason for discontinuation, accounting for 13 of 38 cases (34%). The main side effects leading to cessation were pneumonitis (*n* = 9), colitis/diarrhoea (*n* = 2), fatigue (*n* = 1) and autoimmune disease flare (n = 1). Disease progression was responsible for 11 discontinuations (30%), while other causes included radiation side effects (n = 1), death (n = 2), worsening comorbidities (n = 1), disengagement from oncology care (*n* = 4), and infection (*n* = 3). One patient was retrospectively deemed ineligible following re-review of baseline imaging. Patients who discontinued for reasons other than side effects also experienced treatment-related adverse events, including thyroid dysfunction (n = 2), pruritus (n = 1), adrenal insufficiency (n = 1), and fatigue (n = 1).

Patients who stopped durvalumab due to treatment side effects achieved a median duration of 10 months of treatment, and 53% were still alive at 2 years. The median duration of durvalumab treatment for patients who stopped for other reasons (such as clinical deterioration, disease progression, infection etc) was 9.5 months with 56% still alive at 2 years.

Of the 31 patients (45%) who completed the full course of durvalumab, 17 (55%) developed treatment-related side effects, most commonly pruritus and fatigue/myalgia. Overall, 37 patients (54%) across the entire cohort experienced some form of treatment-related side effect.

## 4 Discussion

Treatment of lung cancer is often complicated by multimorbidity. Smoking, a shared risk factor for lung cancer, chronic obstructive pulmonary disease (COPD) and cardiovascular disease, contributes to the high prevalence of these comorbidities, which increase with age. These complexities influence clinical decision-making, particularly when considering treatments associated with significant side effect burden and complications. This is especially relevant for stage III disease, where larger tumour volumes heighten the risk of treatment-related side effects. Consequently, radiotherapy alone is often prescribed despite the well-established survival benefits of adding systemic anti-cancer therapy (SACT) to radiotherapy [7], [8]. Our findings suggest that the barriers to delivering SACT alongside radiotherapy have persisted despite the introduction of adjuvant immunotherapy.

In this large, multi-MDT cohort study spanning six years, we observed treatment patterns among patients with stage III NSCLC undergoing radical radiotherapy. This availability of durvalumab did not increase overall CRT utilisation but did alter prescribing patterns within SACT. Patients previously treated with sequential CRT are now more likely to receive concurrent CRT. Rates of RT alone have remained unchanged, as did survival outcomes across the study period.

Radiotherapy alone remained the most common treatment across all three study periods, with rates unchanged over time. Compared with patients treated with CRT, those treated with RT were older (median age 72 vs. 64 for concurrent CRT) and had poorer performance status (PS 0: 15% vs. 47%). Interestingly, we found no differences across the different treatment types with regards to comorbidity or deprivation. This suggests that clinician decision making remains subjective, and was not influenced by objective functional assessments in this cohort. These patients experienced significantly higher recurrence rates and increased mortality at two years compared to those receiving radiotherapy combined with SACT and durvalumab. This was independent of age, PS, comorbidities and deprivation. 62% of patients having radiotherapy alone had recurrent (local or distant) disease at 2 years, with 38% of these developing distant metastatic disease. Of those with recurrence, 59% received no further treatment, with 10% receiving immunotherapy, 8% chemotherapy, 3% stereotactic radiosurgery or targeted therapy, and 18% palliative radiotherapy. This pattern suggests that barriers to prescribing chemotherapy alongside initial radiotherapy persist following recurrence. Patients who did receive systemic therapy for distant relapse had better baseline PS (PS 0/1 in 85% vs 75% among those who did not), although age did not differ. Further investigation is needed to clarify these barriers, which include contraindications to platinum chemotherapy related to PS, comorbidities and high tumour burden. Notably, the majority of patients with PS1 in this cohort received radiotherapy alone (63%), which indicated that frailty and comorbidity, factors not fully captured by PS, may influence clinicians decision-making regarding additional systemic treatment. However, formal measurement of patient comorbidity with the CCI in this cohort did not correlate with treatment decisions, suggesting that current tools capturing comorbidity and frailty are not adequate discriminators. It should be noted that a formal assessment of patient frailty was not included in this study due to the retrospective and observational nature of the work.

The PACIFIC trial marked a paradigm shift in the treatment of unresectable stage III NSCLC [2], [9]. PACIFIC-6, published in 2022, further supported the tolerability of sequential CRT followed by durvalumab, although this was not a randomised study [2], [9]. Our real-world analysis demonstrates that the use of concurrent CRT, with or without durvalumab, gradually increased from 2017 to 2022, accompanied by a decline in sequential CRT (Fig. 2). This trend suggests that, following PACIFIC, clinicians have gained confidence in prescribing concurrent CRT for patients previously considered suitable only sequential treatment. Importantly, access to treatment has been optimised through upfront reflex PD-L1 testing, available for 93% of patients since 2019, alongside expanded genetic testing for non-squamous histologies.

This two-year survival analysis in a large real-world cohort confirms the applicability of the PACIFIC trial findings to routine clinical practice. Outside the trial setting, patients treated with concurrent CRT plus durvalumab demonstrated an improved two-year progression free survival compared with concurrent CRT alone, although differences in overall survival were modest and not statistically significant (PFS 64% vs 40%; OS 66% vs 56%). These results mirror the pattern observed in PACIFIC, where PFS benefit translated into a substantial five-year overall survival advantage in the updated analysis [1]. Encouragingly, these benefits were replicated in a population with significant socioeconomic deprivation (62% from the most deprived regions) and multimorbidity (median CCI of 3 (2 - 5)), a group often under-represented in clinical trials [10]. Extended follow-up should clarify whether this advantage translates into a meaningful long-term survival benefit for this population.

Among patients who did not receive durvalumab, sequential CRT appeared to provide similar effectiveness to concurrent CRT, with minimal difference in two-year survival and recurrence outcomes. Notably, these patients were more likely to undergo a subsequent treatment for recurrent disease compared to RT alone (concurrent CRT 75%, sequential CRT 61%, RT alone 39%), which likely contributed to observed differences in OS, particularly given the absence of significant differences in PFS. Rates of immunotherapy at recurrence were substantially higher among patients initially treated with CRT (concurrent CRT 52%, sequential CRT 50%), compared with RT alone (10%).

Previous studies have demonstrated survival advantages for concurrent CRT over sequential CRT [11], [12], [13], [14]; however, modern advances in radiotherapy delivery and systemic therapy may have influenced these findings. Reduced dose intensity during concurrent treatment could also explain the attenuation of these differences. Additionally, patient fitness remains a critical determinant of outcomes as these landmark studies have shown that radiation pneumonitis and other acute side effects are more frequent with concurrent treatment, potentially impacting survival in this real-world population. The outcomes associated with sequential CRT in this cohort must be treated with caution however as the subgroup size is small and therefore underpowered. These data suggest that direct comparisons between concurrent CRT and sequential CRT is warranted, especially given that durvalumab is now licensed in the sequential CRT setting [2].

Treatment with durvalumab in this cohort was challenging, with only 45% of patients completing the planned 12-month course (median duration: 9 months). The discontinuation rate (55%) was substantially higher than reported in the PACIFIC trial (15.5%), likely reflecting the socioeconomically deprived and comorbid population studied, which differs markedly from clinical trial cohorts. Discontinuation rates in other real-world studies vary considerably [9], [15], [16], [17], [18]. In this cohort, treatment-related side effects were common, affecting 54% of patients. Pneumonitis was the leading cause of discontinuation (24%), although the overall spectrum of adverse events was broadly consistent with previous reports.

It remains unclear whether concurrent CRT itself is a direct barrier to receiving immunotherapy. Emerging strategies are exploring different treatment paradigms. The publication of PACIFIC-6, which supports sequential CRT followed by durvalumab, may help mitigate concerns regarding the tolerability of concurrent treatment. This approach could offer an option for patients currently treated with radiotherapy alone, enabling sequential CRT and subsequent durvalumab, available in Scotland but not uniformly across the UK.

Alternative systemic strategies that avoid platinum chemotherapy may also improve the feasibility of concurrent drug-radiotherapy combinations. For example, the CONCORDE study is a phase 1b trial randomising patients to radiotherapy alone or radiotherapy combined with a small molecule DNA damage inhibitor [19], with some participants eligible to receive durvalumab. Additionally, a phase 2 trial has demonstrated acceptable tolerability of durvalumab following radiotherapy alone, with encouraging PFS and OS in patients deemed unsuitable for CRT [20]. However, a randomised trial is required to determine whether immunotherapy confers benefit without chemotherapy in this population.

Of the 31 patients who completed 12 months of durvalumab 77% were disease free at two years. Notably, 53% of patients who discontinued treatment early also achieved disease-free status at two years, suggesting that some individuals may not require the full year of treatment to derive durable benefit. Furthermore, 50% of those who discontinued durvalumab but remained disease-free stopped treatment due to side effects. This observation aligns with published evidence indicating that early cessation of immunotherapy due to adverse events can still result in favourable long term outcomes [21], [22], [23], [24], [25], [26], [27]. These data would suggest that formal evaluation of the relationship between immunotherapy side effects and clinical outcomes is warranted.

This study is limited by its observational design and reliance on real-world treatment decisions rather than randomised allocation. Baseline characteristics varied between treatment modalities, introducing potential confounding factors that, although adjusted for in multivariate regression, cannot be fully eliminated. Due to the retrospective nature of this study, it was not possible to reliably investigate and report why patients receiving concurrent CRT and eligible for durvalumab were not given this treatment. Another key limitation is the restriction of OS data to two years, which precludes assessment of long-term durability of concurrent CRT with durvalumab compared to concurrent CRT alone. However, the 2-year PFS data enables meaningful efficacy comparisons and aligns with outcomes reported in the PACIFIC trial.

Additionally, all patients were treated at a single large cancer centre, which may limit generalisability. Nevertheless, this cohort represents consecutive referrals from multiple multidisciplinary teams across the West of Scotland and reflects a population comparable to other UK and European regions characterised by high levels of social deprivation and multimorbidity in an ageing demographic.

Further work is needed to understand why many patients receive radiotherapy alone, including whether absolute contraindications to platinum chemotherapy are present. Clinical tools that more accurately assess comorbidity and frailty could support decision-making. Certain subgroups may have a low risk of side effects with weekly chemotherapy administered concurrently with radiotherapy. Strategies such as prehabilitation or frailty optimisation may also improve treatment suitability [28], [29], [30]. If poor performance status is primarily driven by lung cancer burden, earlier detection through screening could identify patients more likely to tolerate intensive treatment. However, for many individuals alternative strategies will remain necessary.

## 5 Conclusion

This study demonstrates improved progression-free survival for patients treated with concurrent chemoradiotherapy plus durvalumab in a real-world setting, with outcomes comparable to the PACIFIC trial. Over six years, the use of concurrent chemoradiotherapy increased, suggesting growing confidence in delivering this regime. However, most patients eligible for radical radiotherapy remained unsuitable for additional systemic treatment, which was associated with poorer outcomes. These findings highlight the need for strategies to improve treatment accessibility and optimise care for socioeconomically disadvantaged populations, who are often affected by multimorbidity and frailty.

## CRediT authorship contribution statement

**Adam L. Peters:** Writing – review & editing, Writing – original draft, Software, Methodology, Formal analysis, Data curation, Conceptualization. **Harrison Douglas Stubbs:** Writing – review & editing, Writing – original draft, Formal analysis, Data curation. **Sean F. Duncan:** Writing – review & editing, Writing – original draft. **Audrey Shaw:** Project administration, Data curation. **Karen Moore:** Writing – review & editing, Data curation. **Erin McGarry:** Data curation. **Selina Tsim:** Writing – review & editing. **Graeme Lumsden:** Writing – review & editing. **Mark Stares:** Writing – review & editing, Writing – original draft, Methodology. **Iain Phillips:** Writing – review & editing, Writing – original draft, Methodology. **David Y. Lewis:** Writing – review & editing. **Kevin G. Blyth:** Writing – review & editing, Methodology. **John D. Maclay:** Writing – review & editing, Writing – original draft, Supervision, Methodology, Formal analysis, Data curation, Conceptualization.

## Declaration of competing interest

The authors declare that they have no known competing financial interests or personal relationships that could have appeared to influence the work reported in this paper.
