# Supplementary material for: Real-world outcomes of stage III non-small cell lung cancer in the durvalumab era: insights from a socioeconomically deprived population
Source: Clin Transl Radiat Oncol. 2026 Jul 10;60:101232. doi: 10.1016/j.ctro.2026.101232 (PMC13400429; doi:10.1016/j.ctro.2026.101232)
Supplement: Supplementary file 1 — Supplementary material [file mmc1.docx]

Supplemental Table 1: Patient Demographics by Time Period

|  | **Whole Cohort** | | **Time Period** | | | |
| --- | --- | --- | --- | --- | --- | --- |
| **Variable** | **N** | **N = 656**^1^ | **Pre-Durvalumab**  N = 188^1^ | **Peri-Durvalumab**  N = 252^1^ | **Post-Durvalumab**  N = 216^1^ | **p-value**^2^ |
| **Age at MDT** | 656 | 69 (62 – 75) | 69 (63 – 74) | 69 (62 – 75) | 70 (62 – 75) | 0.94 |
| **Gender** | 656 |  |  |  |  | 0.64 |
| Female |  | 299 (46) | 91 (48) | 111 (44) | 97 (45) |  |
| Male |  | 357 (54) | 97 (52) | 141 (56) | 119 (55) |  |
| **ECOG PS** | 656 |  |  |  |  | **0.002** |
| 0 |  | 182 (28) | 69 (37) | 54 (21) | 59 (27) |  |
| 1 |  | 351 (54) | 92 (49) | 137 (54) | 122 (56) |  |
| 2 |  | 123 (19) | 27 (14) | 61 (24) | 35 (16) |  |
| **Smoking Status** | 656 |  |  |  |  | 0.35 |
| Current |  | 301 (46) | 87 (46) | 120 (48) | 94 (44) |  |
| Ex Smoker |  | 338 (52) | 99 (53) | 126 (50) | 113 (52) |  |
| Non Smoker |  | 17 (2.6) | 2 (1) | 6 (2) | 9 (4) |  |
| **Charlson Comorbidity Index (CCI)** | 656 | 3 (2 – 5) | 3 (2 – 5) | 3 (2 – 5) | 3 (2 – 5) | 0.42 |
| **Scottish Index of Multiple Deprivation (SIMD)** | 656 |  |  |  |  | 0.86 |
| 1 (most deprived) |  | 241 (37) | 78 (41) | 89 (35) | 74 (34) |  |
| 2 |  | 167 (25) | 43 (23) | 68 (27) | 56 (26) |  |
| 3 |  | 108 (16) | 30 (16) | 38 (15) | 40 (19) |  |
| 4 |  | 72 (11) | 18 (10) | 29 (12) | 25 (12) |  |
| 5 (least deprived) |  | 68 (10) | 19 (10) | 28 (11) | 21 (10) |  |
| **Chronic Obstructive Pulmonary Disease (COPD)** | 656 | 100 (15) | 29 (15) | 37 (15) | 34 (16) | 0.95 |
| **Cardiac Disease** | 656 | 55 (8.4) | 19 (10) | 20 (8) | 16 (7) | 0.59 |
| **Diabetes** | 656 | 38 (5.8) | 10 (5) | 14 (6) | 14 (7) | 0.86 |
| **Liver Disease** | 656 | 13 (2.0) | 4 (2) | 2 (1) | 7 (3) | 0.16 |
| **Cerebrovascular Disease** | 656 | 42 (6.4) | 16 (9) | 14 (6) | 12 (6) | 0.38 |
| **Peripheral Vascular Disease (PVD)** | 656 | 34 (5.2) | 10 (5) | 15 (6) | 9 (4) | 0.68 |
| **FEV1/FVC Ratio (%)** | 363 | 63 (55 – 70) | 64 (55 – 71) | 64 (53 – 71) | 63 (56 – 70) | 0.83 |
| **TLCO (%)** | 330 | 64 (52 – 76) | 65 (52 – 77) | 60 (47 – 70) | 65 (53 – 76) | 0.16 |
| **Histology** | 656 |  |  |  |  | 0.44 |
| Adenocarcinoma |  | 203 (31) | 63 (34) | 69 (27) | 71 (33) |  |
| Squamous |  | 342 (52) | 90 (48) | 138 (55) | 114 (53) |  |
| NSCLC other |  | 76 (12) | 21 (11) | 32 (13) | 23 (11) |  |
| No histology |  | 35 (5.3) | 14 (7) | 13 (5) | 8 (4) |  |
| **Stage** | 656 |  |  |  |  | 0.070 |
| IIIA |  | 356 (54) | 113 (60) | 122 (48) | 121 (56) |  |
| IIIB |  | 271 (41) | 71 (38) | 117 (46) | 83 (38) |  |
| IIIC |  | 29 (4.4) | 4 (2) | 13 (5) | 12 (6) |  |
| **PDL1 Status** | 656 |  |  |  |  | **<0.001** |
| <1% |  | 273 (42) | 52 (28) | 119 (47) | 102 (47) |  |
| >50% |  | 117 (18) | 20 (11) | 43 (17) | 54 (25) |  |
| 1-50% |  | 145 (22) | 39 (21) | 62 (25) | 44 (20) |  |
| Unknown |  | 121 (18) | 77 (41) | 28 (11) | 16 (7) |  |
| **ALK/EGFR/ROS1 Status** | 656 |  |  |  |  | **0.020** |
| EGFR positive |  | 6 (0.9) | 2 (1) | 0 (0) | 4 (2) |  |
| EGFR uncertain significance |  | 2 (0.3) | 1 (1) | 1 (1) | 0 (0) |  |
| Negative |  | 228 (35) | 65 (35) | 78 (31) | 85 (39) |  |
| Not tested - SCC |  | 340 (52) | 89 (47) | 138 (55) | 113 (52) |  |
| Unknown |  | 80 (12) | 31 (16) | 35 (14) | 14 (7) |  |
| **Treatment** | 656 |  |  |  |  | **<0.001** |
| RT alone |  | 387 (59) | 111 (59) | 157 (62) | 119 (55) |  |
| Sequential |  | 59 (9.0) | 26 (14) | 21 (8) | 12 (6) |  |
| Concurrent CRT |  | 141 (21) | 47 (25) | 42 (17) | 52 (24) |  |
| Durvalumab |  | 69 (11) | 4 (2) | 32 (13) | 33 (15) |  |
| **RT Dose/Fractionation** | 656 |  |  |  |  | **<0.001** |
| 54Gy/36# (CHART) |  | 140 (21) | 58 (31) | 40 (16) | 42 (19) |  |
| 55Gy/20# |  | 495 (75) | 128 (68) | 210 (83) | 157 (73) |  |
| Other dose |  | 21 (3.2) | 2 (1) | 2 (1) | 17 (8) |  |
| **Time on Adjuvant Durvalumab (months)** | 69 | 9 (2 – 12) | 9 (5 – 12) | 12 (3 – 12) | 6 (2 – 11) | 0.55 |
| **Deceased at 2-years** | 656 | 381 (58) | 111 (59) | 152 (60) | 118 (55) | 0.44 |
| **Lung Cancer Death at 2-years** | 656 | 336 (51) | 105 (56) | 128 (51) | 103 (48) | 0.26 |
| **Recurrence at 2-years** | 656 | 385 (59) | 118 (63) | 144 (57) | 123 (57) | 0.40 |
| **Local Recurrence at 2-years** | 656 | 159 (24) | 57 (30) | 53 (21) | 49 (23) | 0.065 |
| **Distant Recurrence at 2-years** | 656 | 239 (36) | 63 (34) | 91 (36) | 85 (39) | 0.47 |
| ^1^Median (IQR); n (%) | | | | | | |
| ^2^Kruskal-Wallis rank sum test; Pearson's Chi-squared test | | | | | | |
